# Functionalized calcium phosphate nanoparticles to direct osteoprotegerin to bone lesion sites in a medaka (*Oryzias latipes*) osteoporosis model

**DOI:** 10.3389/fendo.2023.1101758

**Published:** 2023-02-22

**Authors:** Nurgul Imangali, Viktoriya Sokolova, Kathrin Kostka, Matthias Epple, Christoph Winkler

**Affiliations:** ^1^ Department of Biological Sciences and Centre for Bioimaging Sciences, National University of Singapore, Singapore, Singapore; ^2^ Inorganic Chemistry and Center for Nanointegration Duisburg-Essen (CENIDE), University of Duisburg-Essen, Essen, Germany

**Keywords:** osteoporosis, nanoparticles, bone resorption, Rankl, osteoprotegerin, OPG

## Abstract

Calcium phosphate (CaP) is the inorganic part of hard tissues, such as bone, teeth and tendons, and has a high biocompatibility and good biodegradability. Therefore, CaP nanoparticles functionalized with DNA encoding bone anabolic factors are promising carrier-systems for future therapeutic development. Here, we analysed CaP nanoparticles in a genetically modified medaka fish model, where osteoporosis-like lesions can be induced by transgenic expression of receptor activator of nuclear factor kappa-B ligand (Rankl). Rankl-transgenic medaka were used to visualize and understand effects of microinjected functionalized CaP nanoparticles during modulation of osteoclast activity *in vivo*. For this, we synthetized multi-shell CaP nanoparticles by rapid precipitation of calcium lactate and ammonium hydrogen phosphate followed by the addition of plasmid DNA encoding the osteoclastogenesis inhibitory factor osteoprotegerin-b (Opgb). An additional layer of poly(ethyleneimine) was added to enhance cellular uptake. Integrity of the synthesized nanoparticles was confirmed by dynamic light scattering, scanning electron microscopy and energy dispersive X-ray spectroscopy. Fluorescently labelled CaP nanoparticles were microinjected into the heart, trunk muscle or caudal fins of Rankl-transgenic medaka embryos that expressed fluorescent reporters in various bone cell types. Confocal time-lapse imaging revealed a uniform distribution of CaP nanoparticles in injected tissues and showed that nanoparticles were efficiently taken up by macrophages that subsequently differentiated into bone-resorbing osteoclasts. After Rankl induction, fish injected with Opg-functionalized nanoparticles showed delayed or absent degradation of mineralized matrix, i.e. a lower incidence of osteoporosis-like phenotypes. This is proof of principle that CaP nanoparticles can be used as carriers to efficiently deliver modulatory compounds to osteoclasts and block their activity.

## Introduction

Bone is constantly remodelled to maintain its rigidity and bone mineral density (reviewed in 1). This requires repeated rounds of bone resorption by osteoclasts that are derived from the monocyte/macrophage lineage. Bone resorption is immediately followed by formation of new bone matrix, which is deposited by osteoblasts that originate from mesenchymal progenitor cells. Osteoclast formation and activity is tightly controlled by a triad consisting of receptor-activator of nuclear kappa-B ligand (Rankl), Rank and osteoprotegerin (OPG). Rankl binds to Rank on the surface of osteoclast precursors to induce differentiation, while osteoblast-derived OPG serves as a secreted decoy receptor that binds to Rankl to prevent its interaction with Rank (2). Formation and activation of osteoblasts and osteoclasts needs to be coordinated to prevent excessive bone resorption and maintain homeostatic bone remodeling. Any disruption results in skeletal diseases such as osteoporosis, a chronic disorder characterized by reduced bone mass, deterioration of bone microarchitecture and fracture-prone bones. Women and men over the age of 50 have an increased risk for osteoporotic fractures, with up to 9 million osteoporotic fractures reported globally (3, 4). Current osteoporosis treatments either use inhibitors of bone resorption or enhancers of bone formation. Anti-resorptive drugs such as bisphosphonates represent a frequently used first-line therapy to reduce resorption by blocking overall osteoclast activity. However, prolonged bisphosphonate exposure reduces bone-cell coupling and impairs bone remodelling to result in more fracture-prone bones (reviewed in 5). Therefore, more targeted therapies are needed that maintain bone quality in a controlled manner especially after long-term use.

Nanoparticles have gained increasing attention as delivery vehicles in therapy, e.g. of cancer (reviewed in 6). They offer deep tissue penetration and efficiently deliver chemicals, growth factors or nucleic acids to target tissues, including bone (reviewed in 7). Historically, calcium phosphate nanoparticles (CaP) were first used as gene carriers in 1973 with reasonable transfection rates *in vitro* (8). The transfection efficiency of triple-shell CaP was shown to be similar to commercially available Polyfect transfection agents and CaP nanoparticles were reported to be safe and non-toxic (9). In a bone context, previous *in vitro* studies suggested that particles could induce synthesis of various cytokines such as Rankl within osteoblasts, which in turn enhanced formation of osteoclasts (10, 11). Nanoparticles were also used to deliver osteoblast-inducing proteins BMP2 and Runx2 to bone matrix, which resulted in increased osteogenesis *in vitro* (12–14)*. In vivo*, a scaffold coated with CaP nanoparticles and implanted into mice released bisphosphonates as well as plasmid DNA encoding BMP2, which both enhanced bone formation (15, 16). Thus, an osteogenic effect of functionalized nanoparticles has been established. However, these approaches so far have not been used under osteoporotic conditions and the efficacy of applied nanoparticles under such conditions remains unknown.

Small fish models have become popular for bone research and modelling human bone disorders, including osteoporosis (17, 18). Our lab previously established a medaka fish osteoporosis model, where inducible expression of Rankl triggered ectopic formation of osteoclasts, excessive bone resorption and osteoporosis-like bone lesions (19, 20). In the present study, we took advantage of translucent medaka larvae and used live confocal imaging to visualize dynamics and distribution of injected CaP nanoparticles under osteoporosis-like conditions. Nanoparticles were functionalized with plasmid DNA encoding mCherry or osteoprotegerin-b (Opgb), and assessing bone cell activity and mineralization revealed a protective effect of functionalized nanoparticles. Our findings suggest an efficient osteomodulatory capacity of functionalized CaP nanoparticles as vehicles *in vivo*.

## Materials and methods

### Fish maintenance and transgenic lines

All animal experiments were performed in accordance with protocols approved by the Institutional Animal Care and Use Committee (IACUC) of NUS (protocol numbers: R14-293, R18-0562, BR15-0119, BR19-0120). Medaka strains were kept in the fish facility at the National University of Singapore (NUS), Department of Biological Sciences (DBS) under a controlled photoperiod of 14 hours light/10 hours darkness at 28°C to induce spawning. Collected embryos were raised in 0.3 x Danieau’s solution (fish medium; 0.23 mM KCl, 19.3 mM NaCl, 0.2 mM Ca(NO_3_)_2_, 0.13 mM MgSO_4_ and 1.7 mM HEPES, pH 7.0) in a 30°C incubator, and staged according to Iwamatsu (21). Fish medium was changed daily and embryos were screened for fluorescence reporter expression from 5 days post fertilization (dpf) onwards. Hatchlings were raised at 30°C until 14 dpf, and 28°C from 14 dpf onwards. Wild-type (Cab) and transgenic medaka lines labelling macrophages (*mpeg1*:mCherry), osteoclasts (*ctsk*:mCherry), osteoblasts (*osx*:GFP) and their precursors (*col10a1*:nlGFP), as well as carrying a heat-shock inducible Rankl transgene are described in **Supplemental Table 1**. Transgenic fish were maintained as homozygous carriers. Double and triple transgenic lines were generated through cross-breeding of single transgenic lines.

### Cloning of plasmids expressing osteoprotegerin-b

For generation of cDNA encoding osteoprotegerin-b (*opgb*; ENSORLT00000006170), total RNA was extracted from wild-type medaka larvae (10 to 15 larvae pooled per biological replicate; 9 to 18 dpf) or adult medaka fins. Total cDNA synthesis was performed using a RevertAid First Strand cDNA Synthesis kit (Thermo Scientific K1621) with oligo(dT)18 primers and 500 ng to 1 μg of total RNA, according to the manufacturer’s protocol. The medaka *opgb* coding sequence (881 bp) was amplified from cDNA using primers opgF (5’-GGGGAATTCCCACCATGACAGTGCTTTACC-3’) and opgR (5’- CAGCAGGCT GAAGTTTGTAGCTGGAAAAATCAAGCTAC-3’). To generate a *mpeg1:opgb*-p2a-EGFP plasmid, a p2a-EGFP-PolyA sequence was amplified from *osx*:*cxcl9l*-p2a-EGFP (20). The *opgb* cDNA and p2a-EGFP-SV40 fragments were fused by overlap extension PCR using primers that introduced a 5’-*EcoRI* site, followed by a Kozak sequence and a 3’-*ApaI* site. The obtained *opgb*-p2a-EGFP-SV40 fragment was digested with *EcoRI* and *ApaI* restriction enzymes and ligated in frame into a *pI-SceI* plasmid containing the *mpeg1* promoter digested with *EcoRI* and *ApaI* (22). The *mpeg1:opgb*-p2a-EGFP plasmid (**Supplementary Figure S1**) allows ectopic expression of *opgb*, linked to EGFP *via* a self-cleaving p2a peptide, in *mpeg1*-expressing macrophages in medaka. Primers used are listed in **Supplementary Table 2**. For a functionality test, the plasmid was injected into medaka embryos at one-cell stage together with *I-Sce-I* meganuclease and hatchlings were imaged to visualize EGFP expression in macrophages in the aorta-mesonephros-gonad region (AGM) from 6 dpf onwards (**Supplementary Figure S2**).

### Preparation and characterization of nanoparticles

Triple-shell CaP nanoparticles were prepared by a co-precipitation from water according to earlier protocols (23). A 0.1 M NaOH solution (p.a.; Merck, Darmstadt, Germany) was used to adjust the pH of aqueous solutions of calcium-L-lactate (18 mmol L^-1^; p.a., Sigma Aldrich, Waltham, MA, USA) and diammonium hydrogen phosphate (10.8 mmol L^-1^; p.a., Merck, Darmstadt, Germany) to 10. Ultrapure water (Purelab ultra instrument, ELGA) with a specific resistivity of 18.2 MΩ was used for all syntheses. For the colloidal stabilization of CaP nanoparticles and additional fluorescent labelling, the Cy5-labelled cationic polymer polyethyleneimine (PEI-Cy5, 2 mg mL^-1^, *M*_w_ = 25 kDa, Surflay Nanotec GmbH, Berlin, Germany) was used. The three solutions described above were rapidly pumped with peristaltic pumps in a volume ratio 5 mL: 5 mL: 7 mL into a glass vessel containing 3.3 mL ultrapure water to obtain CaP/PEI-Cy5 nanoparticles. To synthesize pDNA-functionalized CaP/PEI-Cy5-nanoparticles, two different plasmids were added to this dispersion, respectively, after stirring for 20 min at room temperature. The first plasmid was pDNA-mCherry, encoding mCherry together with a ubiquitously active CMV enhancer (23), to obtain CaP/PEI-Cy5/pDNA-mCherry nanoparticles. The second plasmid was *mpeg1:opgb*-p2a-EGFP to obtain CaP/PEI-Cy5/*mpeg1:opgb*-p2a-EGFP nanoparticles. Plasmid DNA (pDNA) was purified by column purification following the manufacturer’s instructions (Qiagen Plasmid Maxi Kit; Qiagen, Hilden, Germany). Next, an outer silica shell was attached to protect the pDNA from enzymatic degradation. For this, 5 mL of the pDNA-loaded calcium phosphate nanoparticle dispersion was added to a mixture of 25 µL tetraethoxysilane (TEOS, 98%, Sigma Aldrich, CorpWaltham, MA, USA), 50 µL ammonia solution (7.8 wt%, Carl Roth, Karlsruhe, Germany), and 20 mL ethanol (EtOH, p.a., VWR, Darmstadt, Germany) in a round-bottom flask. The resulting dispersion was stirred at room temperature for 16 h. Particles were isolated at room temperature with a 5430/5430 R centrifuge (Eppendorf AG, Hamburg, Germany) at 30,000 rpm for 35 min, followed by redispersion in 5 mL ultrapure water accompanied by 12 s ultrasonication with a sonotrode (UP50H, sonotrode N7, amplitude 70%, pulse duration 0.8 s, Hielscher Ultrasonics GmbH, Teltow, Germany).

Atomic absorption spectroscopy (AAS) was performed with an M-Series AA spectrometer (Thermo Electron Corporation, Waltham, MA, USA) to determine the calcium concentration in a given dispersion. 0.5 mL of the sample was dissolved in diluted HCl. For the size distribution of the nanoparticles, dynamic light scattering (DLS) and for stability studies, zeta potential measurements were performed with a Zetasizer Nano ZS (*λ*= 633 nm, Malvern Nano ZS ZEN 3600, UK). Scanning electron microscopy (SEM) images were taken with an Apreo S LoVac microscope (ThermoFisher Scientific Inc., Waltham, MA, USA) to show the size and shape of the particles. UV-Vis measurements to determine the Cy5 concentration in the dispersion were performed with a Varian Cary 300 bio-spectrophotometer (Agilent Technologies, Santa Clara, California, USA) in a 400 μL Suprasil^®^ quartz cuvette. To determine the pDNA concentration in the particles, a Microvolume UV-Vis spectrophotometer (*λ*= 260 nm, “Nanodrop”, DS-11 FX+, DeNovix, Wilmington, DE, USA) was used by dropping 2 µL of the resulting dispersion (after purification by centrifugation) onto the measurement surface.

For a longer shelf-life, 250 µL of nanoparticle dispersion were mixed with 10 µL of a d-(+)-trehalose solution (20 mg/mL) as cryoprotectant and freeze-dried in a Christ Alpha 2-4 LSC device (Martin Christ GmbH, Osterode am Harz, Germany). The freeze-dried particles were stable, also for shipping. For application of the nanoparticles, they were redispersed in water or cell culture medium (cell experiments) by gentle sonication/vortexing.

### Injection of nanoparticles

Lyophilized CaP nanoparticles were dispersed in water to a final concentration of 100 μM calcium in water solution. The solution was placed on ice and sonicated with a probe sonicator at 70% amplitude for 10 sec twice with a 10 sec interval to disperse agglomerates into individual nanoparticles. Around 300 nL of nanoparticle dispersion was injected into the heart ventricle, the trunk muscle region above the egg yolk extension or between the fin rays of the caudal fin of medaka larvae. Most injections were done at 9 to 13 days post fertilization (dpf; with one exception at 21 dpf), when macrophages, osteoblasts and osteoclasts can be imaged efficiently by confocal microscopy.

### Live fluorescent bone staining

Live staining of mineralized bone matrix was done by immersing medaka hatchlings (12 to 18 dpf) in either Alizarin Complexone solution (ALC; 0.1% in fish medium, Sigma A3882) for two hours or in a Calcein solution (0.01% in fish medium; Sigma C0875) for one hour in the dark at room temperature (RT). Stained hatchlings were rinsed with fish medium three times (15 mins per rinse, at RT) and mounted in 1.5% low melting agarose on a glass bottom petri dish for live fluorescence imaging (488 nm laser/GFP filter for Calcein; 568 nm laser/mCherry filter for ALC).

### Fluorescence imaging

Live fluorescence imaging was performed using a stereomicroscope (Nikon SMZ18) equipped with the NIS-Elements BR 3.0 software or confocal microscopes (Zeiss Meta 500; Olympus FluoView FV3000; Zeiss LSM900). Medaka hatchlings (8 to 23 dpf) were anaesthetized with 0.005% ethyl 3-aminobenzoate methane sulfonate (Tricaine; Sigma MS-222) and mounted in 1.5% low-melting-point agarose on a glass bottom petri dish. Confocal pictures were taken using 405, 488, 543 or 633 nm laser lines for CFP, GFP, mCherry and Cy5 fluorescent signals, respectively. Time-lapse imaging was performed with Olympus FV3000 or Zeiss LSM900 microscopes by imaging the region of interest for 15-20 hours with 5-10 mins intervals. Imaging data were processed using Olympus FV31S-SW 2.1.1.98, Bitplane Imaris 9.0, ImageJ and Adobe Photoshop CC 2018 software.

### Statistical analyses

The numbers of hatchlings with the indicated phenotype were recorded. Results were presented as percentages with mean (SEM) as determined using Prism 7.2. A Two-tailed Student’s t-test was used to compare individual groups and to determine the significance. The level of significance was set as follows: *0.01<P<0.05, **P<0.01, ***P<0.001 and ****P<0.0001.

## Results

### Characterization of CaP nanoparticles

**Figure 1:**
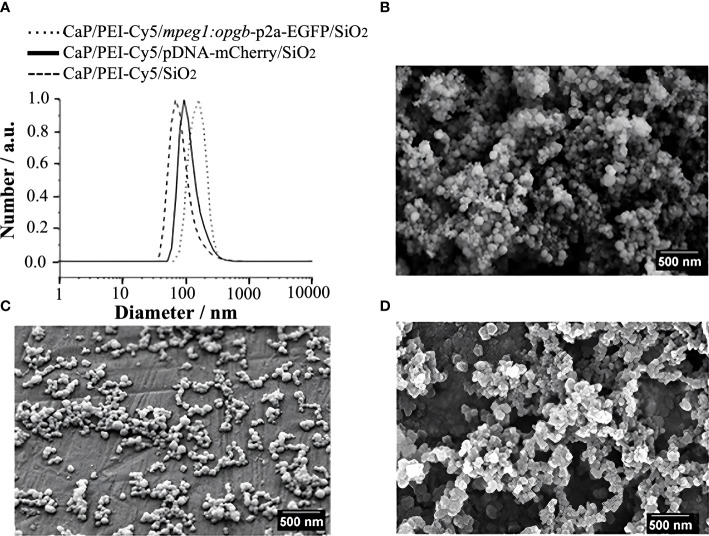
Characterization of CaP nanoparticles. **(A)** Dynamic light scattering (particle size distribution by number) of water-dispersed nanoparticles. **(B)** SEM image of CaP/PEI-Cy5/SiO_2_ nanoparticles. **(C)** SEM image of CaP/PEI-Cy5/pDNA-mCherry/SiO_2_ nanoparticles. **(D)** SEM image of CaP/PEI-Cy5/*mpeg1:opgb-*p2a-EGFP/SiO_2_ nanoparticles. Scale bars = 500 nm.

CaP nanoparticles were synthesised with the fluorescently labelled polymer PEI-Cy5. This allowed the tracking of the particles in biological experiments. The fluorescent polymer was quantified by UV spectroscopy after preparing a calibration row of dissolved PEI-Cy5 (absorption maximum at λ = 670 nm). The hydrodynamic diameter and the stability of the particles were investigated by dynamic light scattering (DLS) (**Figure 1A**). With a polydispersity index (PDI) below 0.3, the dispersion can be considered as well-dispersed. The zeta potential of +20 mV indicated an electrostatic stabilization with a positive charge due to the presence of PEI, which was obviously not compensated by the negative charge of plasmid DNA. Based on the SEM images (**Figures 1B–D**), the morphology of particles was uniform and approximately spherical. **Table 1** summarises all analytical results of CaP/PEI-Cy5/SiO_2_ and CaP/PEI-Cy5/pDNA/SiO_2_ nanoparticles (for extended details on calculations, see 23).

**Table 1:** Characterization of nanoparticles.

Sample	CaP/PEI-Cy5/SiO_2_ M_w_ (PEI-Cy5) = 25.0 kDa	CaP/PEI-Cy5/ pDNA-mCherry/SiO_2_ M_w_ (mCherry) = 28.8 kDa	CaP/PEI-Cy5/ *mpeg1:opgb*-p2a-EGFP/SiO_2_ M_w_ (*opgb*-p2a-EGFP) = 112.1 kDa
Solid core particle diameter by SEM/nm	87 ± 10	88 ± 11	82 ± 15
*V*(one nanoparticle; only CaP)/m^3^	3.45·10^-22^	3.57·10^-22^	2.89·10^-22^
*m*(one nanoparticle; only CaP)/kg	1.08·10^-18^	1.12·10^-18^	9.06·10^-19^
*w*(Ca^2+^) in the dispersion by AAS/kg m^-3^	0.099	0.079	0.081
*w*(Ca_5_(PO_4_)_3_OH) in the dispersion/kg m^-3^	0.248	0.198	0.203
*N*(nanoparticles) in the dispersion/m^-3^	2.29·10^+17^	1.77·10^+17^	2.24·10^+17^
*w*(pDNA) in the dispersion/kg m^-3^	–	0.141 (94%)	0.132 (98%)
*N*(pDNA)/m^-3^	–	2.95·10^+20^	7.09·10^+19^
m(pDNA) per nanoparticle in the dispersion/kg	–	7.97·10^-20^	5.89·10^-20^
*N*(pDNA) molecules per nanoparticle	–	1.67·10^+3^	3.16·10^+2^
*w*(PEI-Cy5) in the dispersion/kg m^-3^	1.60·10^-2^	1.33·10^-2^	1.04·10^-2^
*N*(PEI-Cy5)/m^-3^	3.85·10^+20^	3.20·10^+20^	2.51·10^+20^
*m*(PEI-Cy5) per nanoparticle in the dispersion/kg	6.98·10^-20^	7.52·10^-20^	4.64·10^-20^
*N*(PEI-Cy5) molecules per nanoparticle	1.68·10^+3^	1.81·10^+3^	1.12·10^+3^
Hydrodynamic particle diameter by DLS/nm (number)	68	93	159
Polydispersity index (PDI) by DLS	0.3	0.2	0.4
Zeta potential by DLS/mV	+22	+20	+18

### Nanoparticles are stable after injection into medaka embryos

**Figure 2:**
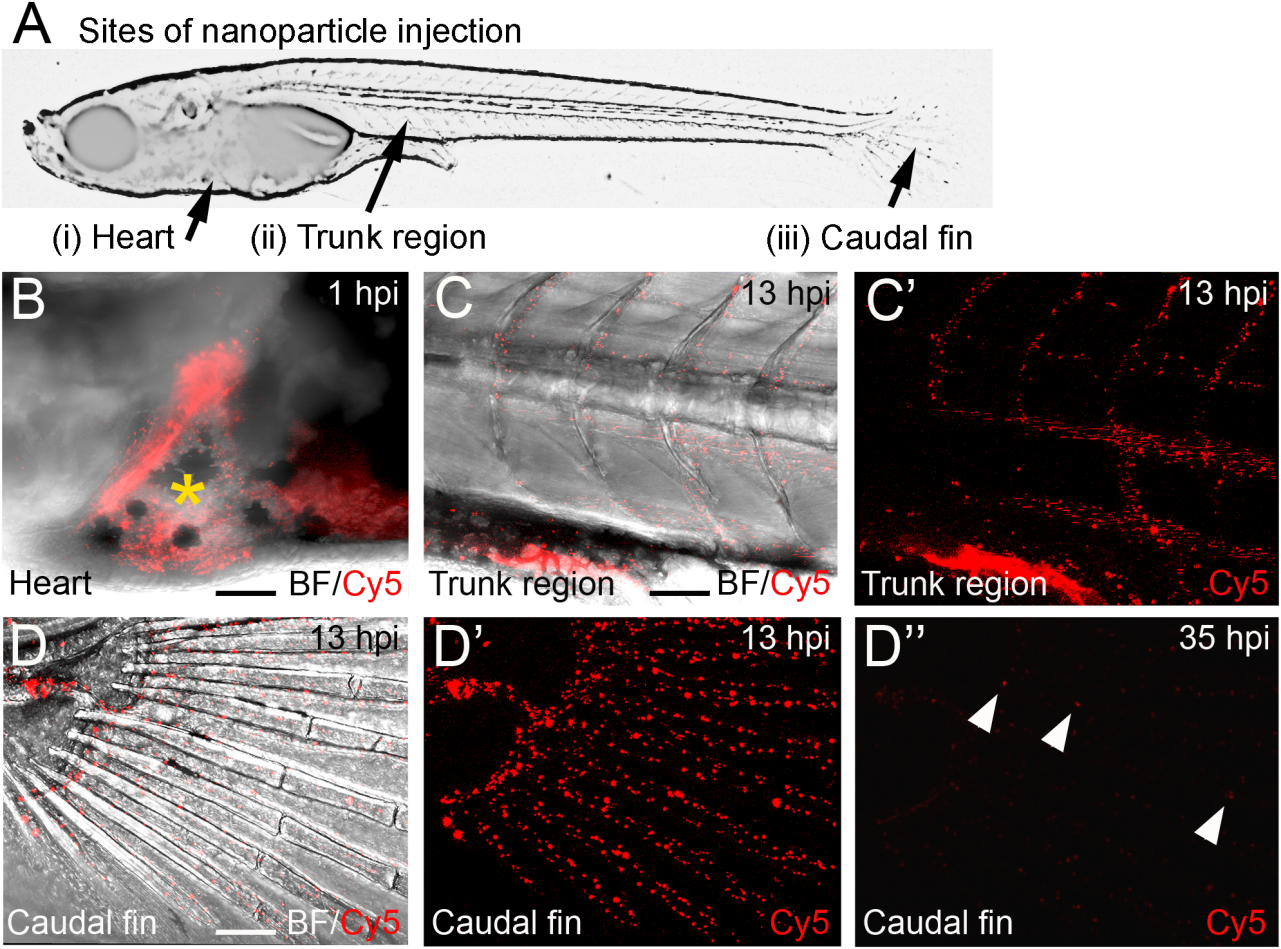
Distribution of Cy5-labelled calcium phosphate nanoparticles after injection into medaka larvae. **(A)** Schematic image of a medaka embryo at 11 days post fertilization (dpf) indicating sites of nanoparticle injection, i.e. heart, muscle fiber region in the rostral trunk and between bony rays of the caudal fin. **(B)** Merged image of brightfield (BF) and Cy5 channel of larval medaka heart at 1 hour post injection (hpi). Cy5 (red) indicates nanoparticles around injection site marked by yellow asterisk. **(C, C’)** After heart injection, Cy-5-labeleld nanoparticles are detectable in blood vessels of the trunk at 13 hpi. A merge of brightfield and fluorescence image **(C)** and the fluorescence image alone are shown **(C’)**. **(D, D”)** After heart injection, Cy5-labelled nanoparticles are detectable in blood vessels of the caudal fin at 13 hpi **(D- D’)** and show strongly reduced fluorescence at 35 hpi **(D”)**. Arrowheads mark remaining fluorescently labeled particles. Ten embryos were analysed and showed this phenotype. Scale bars = 90 μm.

CaP nanoparticles were microinjected into medaka larvae at different stages (8 to 21 days post fertilization, dpf) into the following regions: i) the heart ventricle, ii) into the trunk muscle region positioned next to the aorta-gonad-mesonephros (AGM; a region where macrophages reside that differentiate into osteoclasts after Rankl induction (22)), and iii) between the bony fin rays of the caudal fin (**Figure 2A**). The stability of CaP nanoparticles was assessed by injection of Cy5-labelled nanoparticles into the heart ventricle and subsequent live fluorescent imaging of blood vessels at 1, 13 and 35 hours post injection (hpi). At 1 hpi, nanoparticles were observed in the heart (**Figure 2B**), and at 13 hpi in blood vessels of the trunk (**Figure 2C, C’**), and fin (**Figure 2D, D’**). This pattern suggested that nanoparticles distributed efficiently throughout the whole body and persisted in dispersed form. Nanoparticles were evident in the bloodstream for up to 35 hpi but the fluorescent signal was strongly reduced by this stage (**Figure 2D**”). This showed that injected nanoparticles are stable for at least 35 hours. This time window thus appeared optimal for assessing whether plasmid-functionalized nanoparticles are able to transfect tissue cells.

### Nanoparticles are internalized by osteoclasts and macrophages in the caudal fin

To determine whether nanoparticles are internalized by medaka bone cells, nanoparticles were functionalized with an mCherry reporter plasmid (CaP/PEI-Cy5/pDNA-mCherry/SiO_2_) and injected into transgenic medaka larvae that expressed the following bone cell reporters: *col10a1*-nlGFP in osteoblast progenitors (24), *osx*-GFP in pre-mature osteoblasts (25), and *ctsk*-mCherry in osteoclasts (26).

**Figure 3:**
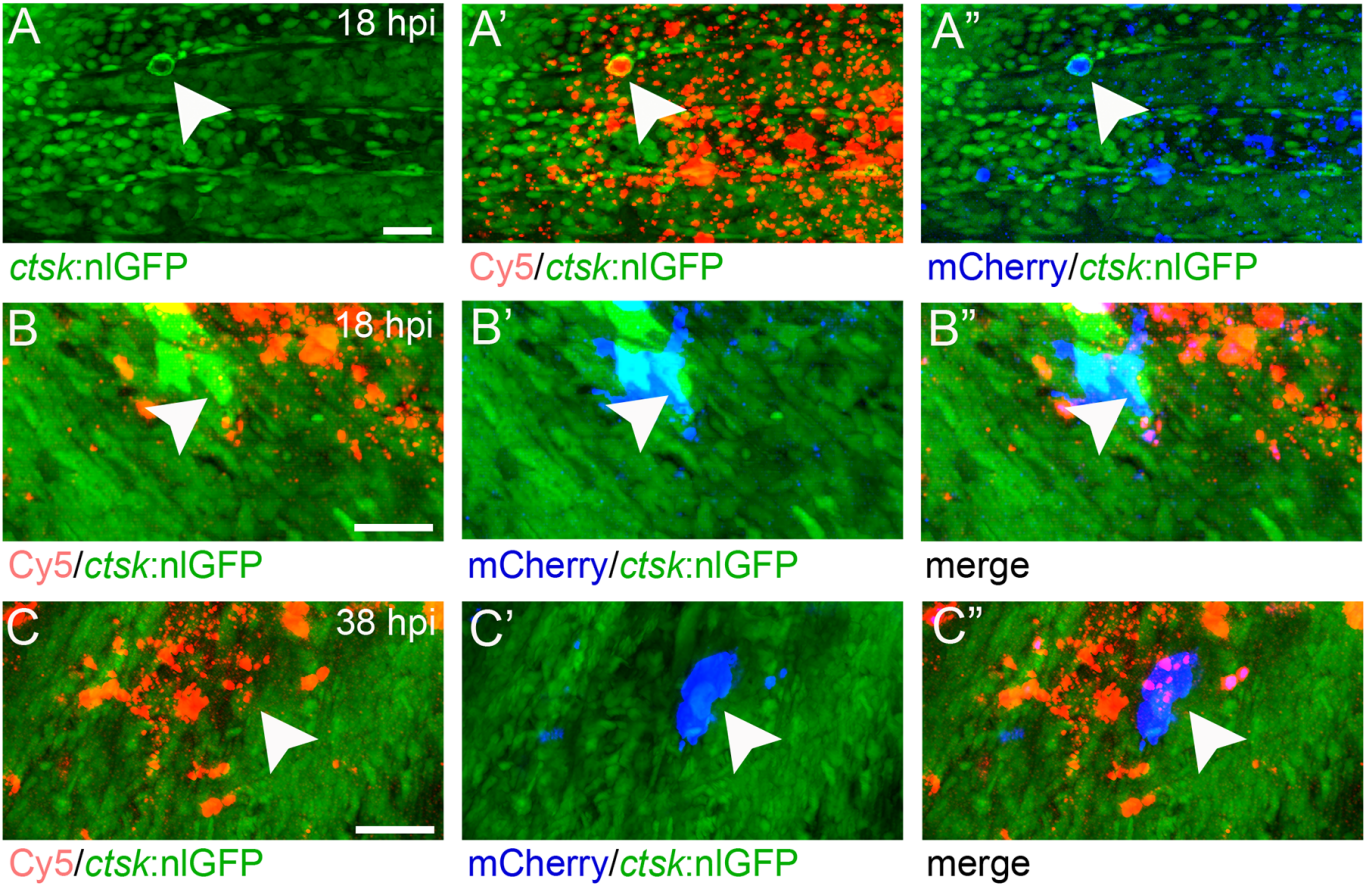
*ctsk*:nlGFP positive cells in the medaka caudal fin internalize plasmid-functionalized CaP nanoparticles and express mCherry protein. **(A, A”)** Caudal fin of *ctsk*:nlGFP transgenic medaka at 12 dpf and 18 hours after injection of CAP/PEI-Cy5/pDNA-mCherry/SiO2 nanoparticles into the fin. Panels show *ctsk*:nlGFP positive cells **(A)**, colocalization of nlGFP and Cy5 **(A’)**, and colocalization of nlGFP and plasmid-encoded mCherry **(A”)**; arrowhead). **(B, C”)** Large and dynamic mCherry expressing cell (arrowheads) at 18 hpi **(B, B”)** and 38 hpi **(C, C”)**. Panels show nlGFP positive cells colocalized with Cy5 **(B, C)**, colocalization of nlGFP with plasmid-encoded mCherry **(B’, C’)** and merge **(B”, C”)**. Cy5 marks nanoparticles (red) and mCherry (blue) marks cells that took up nanoparticles and express reporter protein. 6 out of 10 analysed embryos showed this phenotype. Scale bars = 20 µm **(A)**.

**Figure 4:**
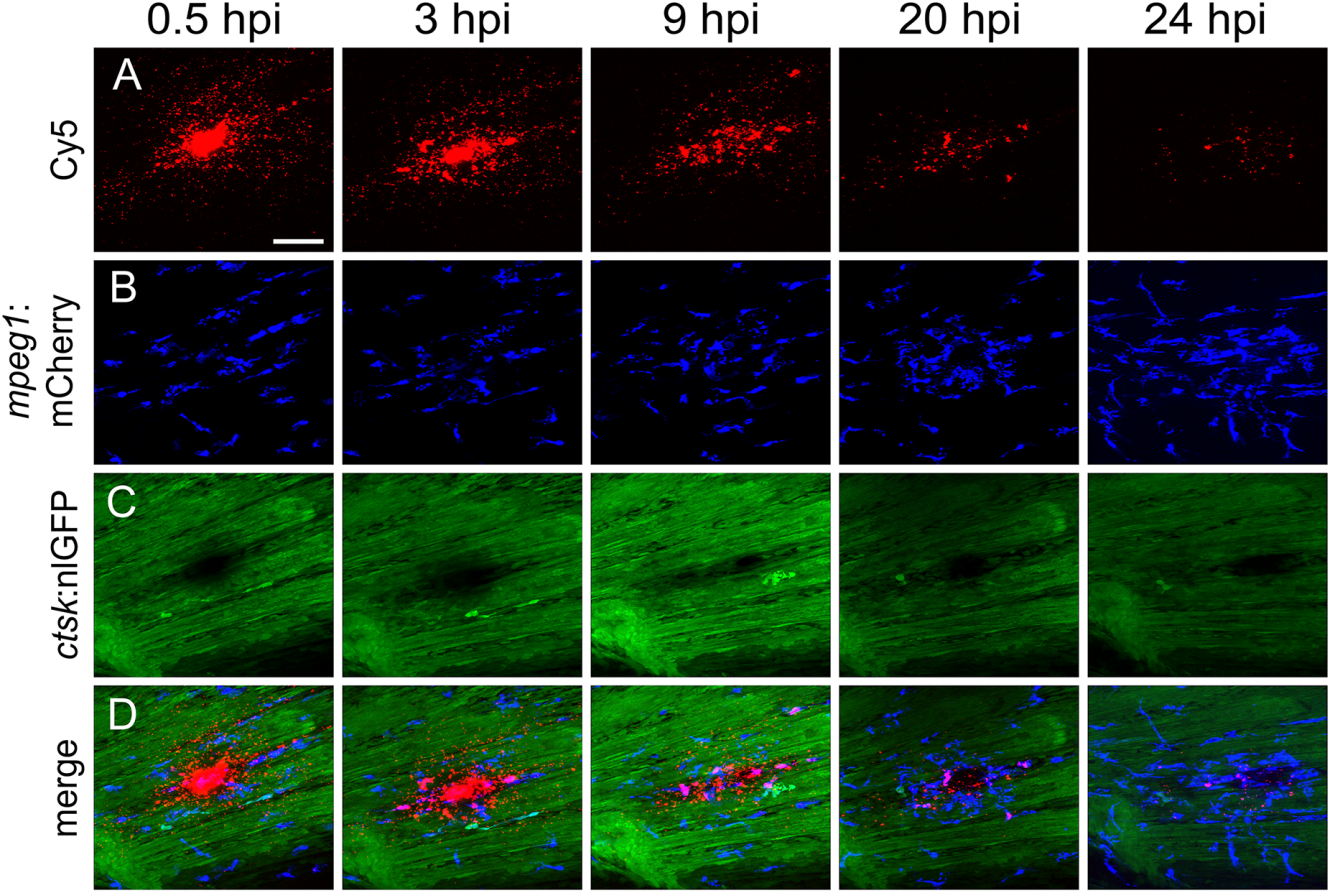
Macrophages are recruited to the injection site and internalize CaP nanoparticles within 24 hours. Cy5-labelled nanoparticles were injected into the caudal fin of *mpeg1*:mCherry/*ctsk*:nlGFP double transgenic medaka larvae at 12 dpf. **(A-D)** Lateral views of a caudal fin showing Cy5-labelled nanoparticles **(A)**, *mpeg1*:mCherry positive macrophages **(B)**, *ctsk*:nlGFP positive osteoclasts **(C)** and merged image **(D)** at 0.5, 3, 9, 20 and 24 hours post injection (hpi), respectively. Scale bar = 40 μm. 3 out of 3 analysed fish showed this phenotype.

First, nanoparticles were injected into the caudal fin in close proximity to bony fin rays. Interestingly, only sparse, if any, colocalization of Cy5 or mCherry from functionalized nanoparticles with signals for *col10a1:nlGFP* or *osx:GFP* could be observed with live fluorescent imaging (**Supplementary Figures S3**, **S4**). Thus, osteoblast progenitors (*col10a1*) and pre-mature osteoblasts (*osx*) are unlikely to efficiently internalize CaP nanoparticles. Importantly, however, mCherry reporter expression was detected in some distinct non-osteoblast cells in the caudal fin as early as 5 hpi (**Supplementary Figures S3**, **S4**) and remained stable at 18 hpi (**Figures 3A, A”, B, B”**) and 38 hpi (**Figure 3C, C”**). Most of the cells that had taken up nanoparticles and expressed mCherry reporter were small and round-shaped and were localized on the outer surface of the bony fin rays (**Figure 3A**; **Supplementary Figures S3**, **S4**). Some of these cells were *ctsk*:nlGFP positive and showed a colocalization of Cy5 and mCherry with nlGFP signals, indicating that they had taken up the functionalized nanoparticles and expressed the introduced plasmid reporter (**Figures 3A, A”**; arrowheads). Some of the *ctsk*:nlGFP cells that had internalized nanoparticles were large and dynamic (**Figures 3B, C”**, arrowheads). Based on their morphology and migratory behaviour, we hypothesized that these cells are macrophages that had switched on the *ctsk*:nlGFP reporter. To test this, nonfunctionalized Cy5 labelled nanoparticles (CAP/PEI-Cy5/SiO_2_) were injected into the caudal fin of *mpeg1*:mCherry transgenic medaka larvae and fluorescent reporter expression was analyzed over 24 hours (**Figure 4**). After injection, a colocalization of nanoparticle derived Cy5 and the macrophage reporter mCherry was observed as early as 3 hpi, which confirmed internalization of nanoparticles by *mpeg1* positive macrophages. Live fluorescence time-lapse imaging showed that mCherry macrophages were recruited to the injection site in the caudal fin and efficiently phagocytosed nanoparticles within 24 hpi, which resulted in a depletion of Cy5 signal over time (**Figure 4**; **Supplementary Movie 1**). These anti-inflammatory *mpeg1*:mCherry macrophages were recruited in response to tissue damage induced by injection, as they were also detected after injection of water as control (**Supplementary Figure S5**).

### Macrophages internalize nanoparticles and differentiate into osteoclasts along the vertebral column

**Figure 5:**
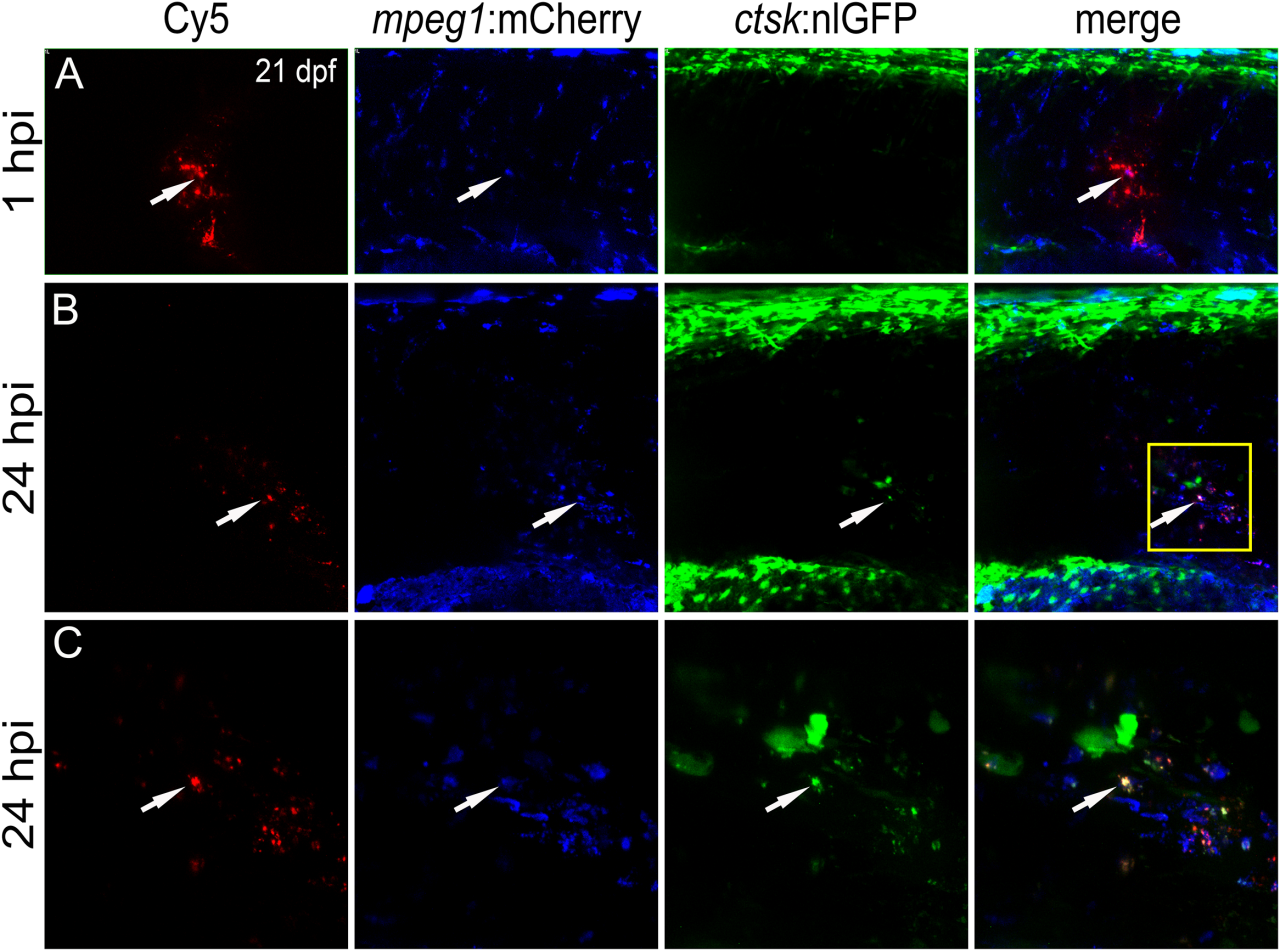
CaP nanoparticles are internalized by Rankl-induced macrophages that subsequently differentiate into osteoclasts. Cy5-labelled CaP nanoparticles were injected into trunk muscle of *mpeg1*:mCherry/*ctsk*:nlGFP/*rankl*:*HSE:*CFP transgenic medaka larvae at 21 dpf after a heat shock had been applied at 20 dpf to induce Rankl expression. **(A)** Lateral view of trunk region showing Cy5-labelled nanoparticles, *mpeg1*:mCherry positive macrophages, *ctsk*:nlGFP positive osteoclasts and merged image at 1 hpi. **(B)** Same larva at 24 hpi. **(C)** High magnification view of region indicated as box in **(B)** Colocalization of Cy5, mCherry and/or nlGFP signals is indicated by white arrowheads. 5 out of 5 analyzed embryos showed this phenotype.

Next, non-functionalized Cy5 nanoparticles were injected into the muscle region above the AGM and close to the vertebral column of *rankl*:*HSE:*CFP/*mpeg1*:mCherry/*ctsk*:nlGFP transgenic fish (**Figure 5**). These fish report the presence of *mpeg1* macrophages in the AGM, as well as their differentiation into *ctsk* positive osteoclasts upon heat-shock-induced Rankl expression (22).

Live imaging revealed that nanoparticle injection after Rankl induction caused most macrophages to migrate from the AGM to the injection site (**Figure 5A**). Recruited as well as resident macrophages at the injection site were found to take up Cy5-labelled nanoparticles and to differentiate into *ctsk:*nlGFP positive osteoclasts (**Figures 5B, C**; **Supplementary Movie 2**). Importantly, the newly differentiated *ctsk* positive osteoclasts retained Cy5 signal (**Figures 5B, C**, arrowheads) indicating that the non-functionalized nanoparticles did not interfere with differentiation of Rankl-induced macrophages. In summary, while osteoblast progenitors and pre-osteoblasts and most of the *ctsk* positive cells were found to not take up injected CaP nanoparticles, *mpeg1* positive macrophages efficiently internalized non-functionalized and functionalized nanoparticles. Upon injection, these macrophages were efficiently recruited to injection sites, internalized nanoparticles and then differentiated into *ctsk* osteoclasts upon Rankl induction. Thus, CaP nanoparticles are potent delivery vehicles to osteoclast progenitors in medaka.

### Nanoparticles deliver osteoprotegerin-b to medaka bone matrix

**Figure 6:**
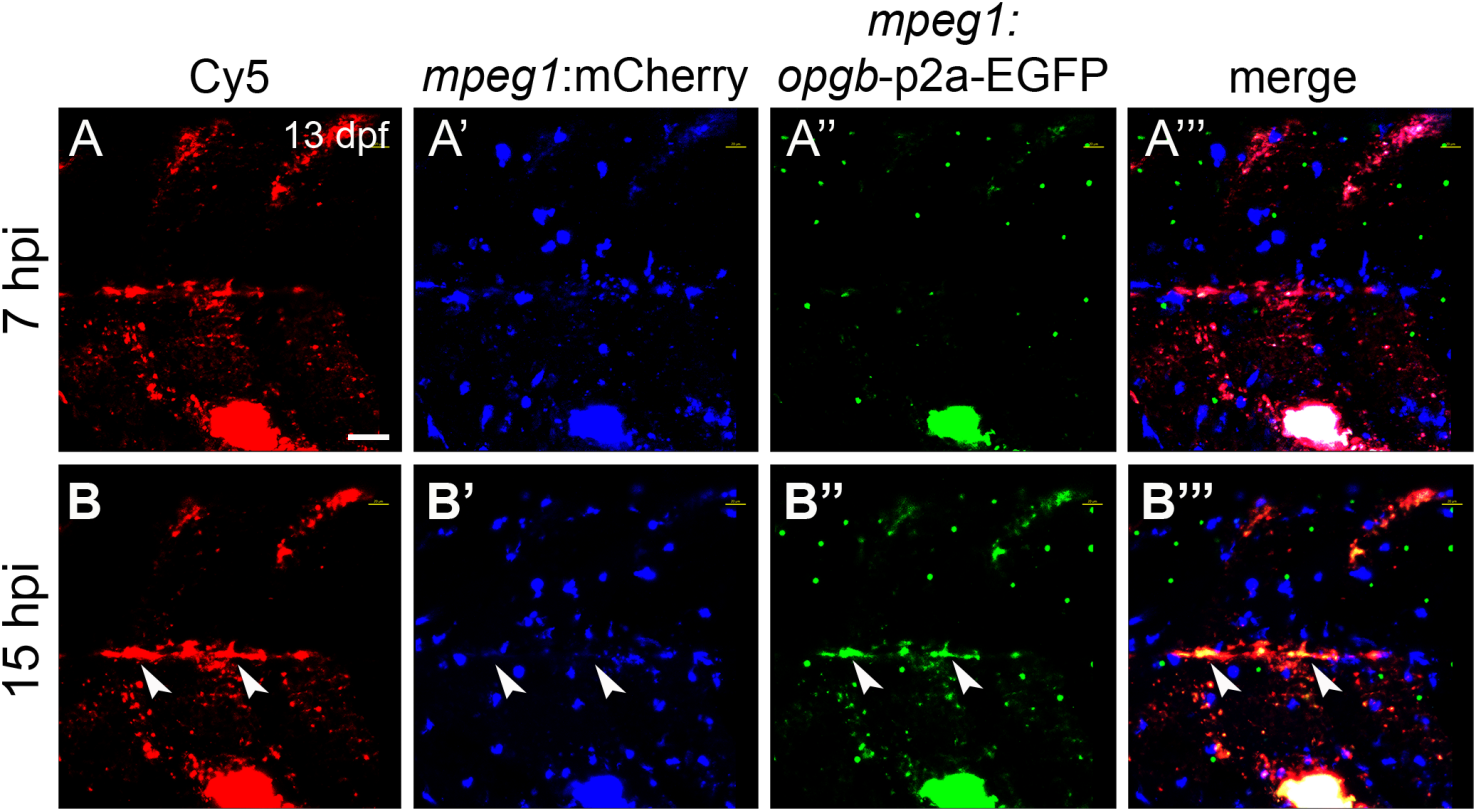
CaP nanoparticles deliver *mpeg1:opgb-*p2a-EGFP plasmid to macrophages. Cy5-labelled CaP nanoparticles functionalized with *mpeg1:opgb-*p2a-EGFP plasmid were injected into trunk muscle above the yolk extension of *mpeg1*:mCherry transgenic larvae at 13 dpf. **(A, A’”)** Lateral view of trunk region at 7 hpi showing Cy5-labelled nanoparticles **(A)**, *mpeg1*:mCherry-expressing macrophages (A’), *mpeg1*:*opgb-*p2a-EGFP expressing cells **(A”)** and a merged image **(A’”)**. Note that some macrophages internalized CaP nanoparticles but did not express *opgb-*p2a-EGFP at this early stage. **(B, B’”)** Same larva at 15 hpi. Note colocalization of Cy5, *mpeg1*:mCherry and *mpeg1:opgb-*p2a-EGFP (arrowheads), suggesting that macrophages have internalized CaP nanoparticles and express *opgb-*p2a-EGFP. Scale bar = 40 μm. 6 out of 6 analyzed embryos showed this phenotype.

Next, nanoparticles were functionalized with *mpeg1:opgb*-p2a-EGFP plasmid to express Opgb specifically in macrophages. Successful transfection of macrophages was monitored by expression of EGFP, which was linked to Opgb through a self-cleavable p2A peptide (**Supplementary Figures S1**, **S2**). Functionalized nanoparticles were injected into the trunk muscle region close to the vertebral column of *mpeg1*:mCherry transgenic fish at 13 dpf. At 7 hpi, macrophages had taken up nanoparticles but did not yet express detectable levels of *opgb*-p2a-EGFP (**Figure 6A**). At 15 hpi, however, several *mpeg1*:mCherry macrophages had a Cy5 label, demonstrating presence of internalized nanoparticles, and. co-expressed mCherry and EGFP (**Figure 6B, B”‘**, arrowheads). This suggested successful expression of CaP-delivered Opgb in these macrophages. Unexpectedly, EGFP signal was also detected outside macrophages suggesting either a possible leakage of the *mpeg1* promoter or inefficient cleavage of the p2A peptide resulting in secretion of EGFP-tagged Opgb.

**Figure 7:**
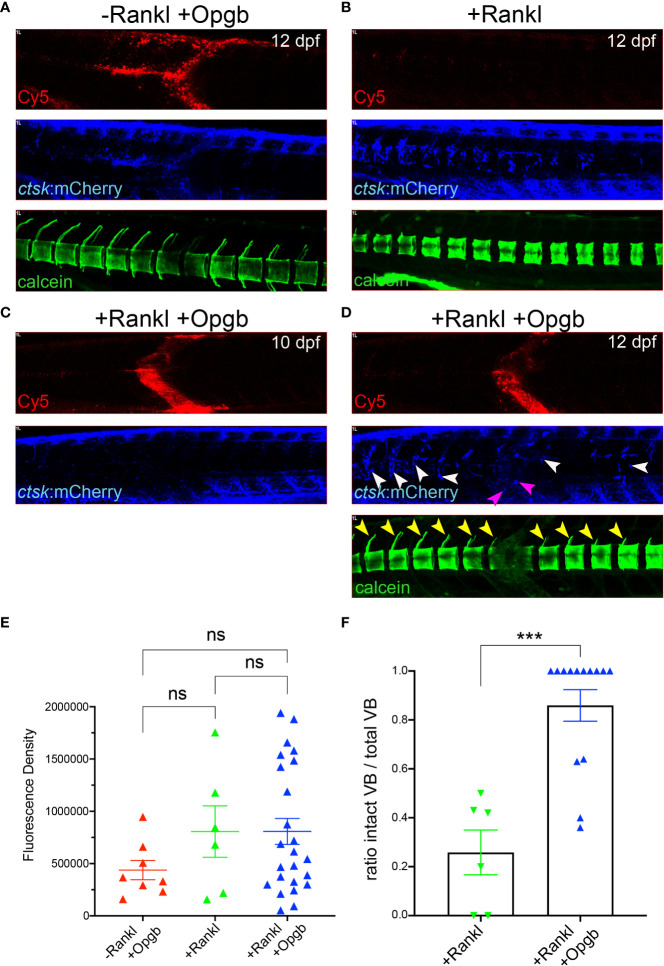
Nanoparticle-derived *opgb*:GFP protects mineralized vertebral arches from resorption by Rankl-induced osteoclasts. Cy5-labelled CaP nanoparticles functionalized with *mpeg1:opgb-*p2a-EGFP plasmid were injected into trunk muscle of *ctsk*:nlGFP/*rankl*:*HSE:*CFP double transgenic larvae at 9 dpf and imaged at 10 and 12 dpf. **(A)** Lateral view of trunk region. In the absence of Rankl induction, Cy5-labelled nanoparticles (top) recruit *ctsk*:mCherry positive cells to the injection site at 72 hpi (middle). Calcein staining at 12 dpf indicates normal mineralization of vertebral arches (bottom, n=4). **(B)** After Rankl induction at 9 dpf and in the absence of CaP nanoparticle injection (top), *ctsk*:mCherry expressing osteoclasts form along vertebral bodies (middle). Calcein staining indicates complete resorption of neural vertebral arches (bottom). **(C)** After Rankl induction at 9 dpf and injection of *mpeg1:opgb-*p2a-EGFP-functionalized nanoparticles at 1 hour after Rankl induction, no *ctsk*:mCherry cells are recruited at 10 dpf (i.e., 24 hpi). **(D)** Same larva as in C at 72 hpi showing formation of Rankl-induced ectopic *ctsk*:mCherry osteoclasts. Calcein staining indicates that despite abundant osteoclasts, mineralization of vertebral neural arches is preserved (n=14 larvae). **(E)** Statistical analysis of relative fluorescence density of mCherry signal at 12 dpf within specified region of interest at injection area, i.e. vertebral bodies (VBs) 8-10, indicative for recruitment of *ctsk* positive cells. **(F)** Statistical analysis of the ratio of calcein-stained VBs with neural arches present over total number of VBs. Only VBs with *ctsk* positive osteoclasts present after Rankl induction were counted. ***, p<0.005; ns, not significant.

Next, we addressed whether the expressed Opgb was functional and could modulate excessive bone resorption triggered by Rankl induction. For this, *rankl:HSE:*CFP/*ctsk*:mCherry double transgenic medaka larvae were heat-shocked at 9 dpf to induce Rankl induction. At 1 hour after Rankl induction, larvae were then injected with *mpeg1:opgb*-p2a-EGFP nanoparticles and then analyzed at 10 and 12 dpf (**Figure 7**). Nanoparticle injected larvae without heat shock (-Rankl, +Opgb) and Rankl-induced larvae without injection of nanoparticles (+Rankl) were used as controls (**Figures 7A, B**). Injection of *opgb*-functionalized nanoparticles resulted in an increased recruitment of *ctsk* positive cells to the injection site even without Rankl induction (**Figure 7A**). The small rounded morphology and dynamic behavior of these recruited cells suggested that these were activated macrophages that had turned on the *ctsk* reporter transgene in response to CaP without fully differentiating into osteoclasts. Live bone staining with calcein showed normal bone mineralization without Rankl induction and no enhanced lesions as in Rankl-induced larvae, despite the presence of *ctsk*:mCherry cells. This supported the idea that recruited *ctsk* positive cells had not differentiated into activated osteoclasts (**Figure 7A**). In contrast, heat shock-induced Rankl expression in control larvae that had not been treated with nanoparticles resulted in increased osteoclast differentiation at vertebral bodies, which led to excessive bone resorption and absent neural and hemal arches as reported earlier (**Figure 7B**; see 19, 20). In larvae injected with nanoparticles after Rankl induction, the recruitment of *ctsk* positive cells started at 24 hpi (10 dpf) and had increased by 72 hpi (12 dpf; **Figure 7C, D**). At 12 dpf, some *ctsk* positive cells were recruited to the injection site and took up nanoparticles (**Figure 7D**, magenta arrowheads), while the majority of newly differentiated *ctsk* positive osteoclasts remained at the vertebral bodies (**Figure 7D**, white arrowheads; **Supplementary Figure S6**). The average fluorescent density of mCherry marking *ctsk* positive cells at the injection site was not significantly different among the three groups (**Figure 7E**). Importantly, neural arches and vertebral centra were largely preserved after Rankl induction in larvae injected with *opgb*-functionalized nanoparticle, which was evident by live bone staining with calcein at 12 dpf (**Figure 7D**, neural arches marked with yellow arrowheads). Statistical analysis showed that compared to Rankl-induced larvae without nanoparticles, the nanoparticle-injected larvae had significantly more vertebral bodies with evident neural arches after Rankl induction (**Figure 7F**). This suggests that although Opgb delivered by nanoparticles did not reduce the density of *ctsk* positive cells (macrophages or osteoclasts), it protected bone integrity from Rankl-induced resorption. In conclusion, we show that CaP nanoparticles functionalized with *opgb-*expressing plasmids were internalized by macrophages in the presence or absence of Rankl induction. Expression of the delivered plasmid protected mineralized bone from Rankl-induced osteoclasts and excessive resorption. This suggests that CaP nanoparticles can act as efficient delivery vehicles of bone-modulating compounds in medaka *in vivo*.

## Discussion

Calcium phosphate (CaP) nanoparticles are regarded as safe and efficient carriers of biomolecules across cell membranes *in vitro* and *in vivo* (9, 27, 28) Their promising potential for bone repair was demonstrated when a nanoparticulate CaP-containing paste was used to successfully deliver osteogenic growth factors to bone in rabbits (28). Compared to other transport vehicles, such as titanium oxide, CaP nanoparticles exhibit no toxic effects but may increase intracellular calcium levels. Hence, high concentrations and prolonged treatment with standard CaP nanoparticles, especially in their non-functionalized form, may cause particle aggregation and accumulation of calcium (29). Accordingly, we previously demonstrated that CaP nanoparticles internalized by HeLa cells did not affect cell proliferation and viability (23) but were degraded in lysosomes within 3 hours after uptake (27).

In the present study, we assessed the effect of CaP nanoparticles *in vivo* in a transgenic medaka model expressing various fluorescent bone cell reporters. We found that non-functionalized (CaP/PEI-Cy5/SiO2) and functionalized (CaP/PEI-Cy5/pDNA-mCherry/SiO_2_; CaP/PEI-Cy5/*mpeg1*:*opgb*-p2a-EGFP/SiO_2_) nanoparticles did not affect the viability of nanoparticle-treated larvae. Cy5-labelled non-functionalized CaP nanoparticles were stable and circulated in the bloodstream for up to 35 hours after injection into the heart of medaka larvae. This suggested that CaP nanoparticles can be used as safe delivery vehicles to medaka bone cells *in vivo*.

### Medaka macrophages internalize CaP nanoparticles

CaP nanoparticles were shown to enter cells *via* macropinocytosis and their internalization depended on the nanoparticle morphology, size and coating (27). The CaP nanoparticles used in the present study were spherical, less than 100 nm in diameter and coated with silica to prevent agglomeration (30). All these properties are known to improve uptake by cells (31). While oral administration of nanoparticles results in high absorption in the intestine (32), the injection of nanoparticles in this study allowed a more targeted delivery to bone cells positioned along the vertebral column and bony fin rays. To assess which type of bone cell internalized the nanoparticles, Cy5-labeled and pDNA:mCherry-functionalized CaP nanoparticles (CaP/PEI-Cy5/pDNA-mCherry/SiO_2_) were injected into transgenic medaka expressing different fluorescent reporters in osteoblast progenitors, pre-osteoblasts, macrophages or osteoclasts. Although injections were done in close proximity to bone and bone cells, very sparse if any uptake of nanoparticles by *col10a1*:nlGFP osteoblast progenitors and *osx*:mGFP pre-osteoblasts was detectable. Our *in vivo* findings are in contrast to several *in vitro* studies that showed that inorganic nanoparticles including hydroxyapatite-based nanoparticles and CaP nanoshells were efficiently internalized by mammalian pre-osteoblasts and osteoblasts and stimulated their differentiation, proliferation and activities (33–40). Future studies are therefore needed to better understand whether a particular differentiation state of osteoblasts is needed *in vivo* to allow uptake of CaP nanoparticles.

Osteoclasts and their macrophage/monocyte progenitors efficiently phagocytose biological particles. Consistent with this, Cy5-labelled CaP nanoparticles were rapidly internalized by *ctsk*:nlGFP expressing cells in the caudal fin. Injection of CaP nanoparticles into muscle tissue also induced an increased density of *ctsk*:nlGFP cells that took up nanoparticles. Based on their morphology and dynamics, as evident by live imaging, these *ctsk*:nlGFP cells most likely represented macrophages that had initiated differentiation into osteoclasts possibly as a response to elevated CaP levels at the injection site. While some inorganic nanoparticles, such as gold nanoparticles, were shown to inhibit osteoclastogenesis *in vitro* (41), CaP nanoparticles might induce differentiation of recruited macrophages into *ctsk*-expressing cells in medaka. To test this hypothesis, Cy5-labelled CaP nanoparticles (CaP/PEI-Cy5/SiO_2_) were injected into *mpeg1:*mCherry transgenic medaka that exhibit fluorescently labelled pro-inflammatory (M1) macrophages (22). This confirmed that *mpeg1*:mCherry macrophages were recruited to injection sites and efficiently internalized CaP nanoparticles within a few hours after injection. How these macrophages internalized several Cy5-labelled particles, however, remains unclear. *In vitro*, human macrophages were shown to take up large amounts of hydroxyapatite or other nanoparticles using a highly branched membranous compartment (the surface connected compartment, SCC) with several openings to the extracellular space (42). Whether a similar mechanism is responsible for nanoparticle internalization in medaka *in vivo* remains to be tested.

Most immature and non-activated *mpeg1*:mCherry macrophages in medaka reside within the aorta-gonadal mesonephros (AGM). In response to Rankl, they relocate to the vertebral column, where they differentiate into *ctsk*:nlGFP expressing osteoclasts (22). In this study, Cy5-labeled nanoparticles (CaP/PEI-Cy5/SiO_2_) were injected close to the medaka AGM after Rankl induction. We found that approximately 30-50 of the Rankl-induced *mpeg1*:mCherry macrophages accumulated at the injection site and internalized nanoparticles. Afterwards, these macrophages differentiated into *ctsk*:nlGFP expressing osteoclasts and retained Cy5 signal within their cytoplasm. This strongly suggests that internalized CaP nanoparticles do not interfere with osteoclast differentiation. Together, our study showed that pro-inflammatory medaka macrophages were recruited to sites of nanoparticle injection, efficiently internalized CaP nanoparticles and differentiated into actively resorbing osteoclasts in response to Rankl induction. This opens the possibility to use nanoparticles as vehicles to introduce bone modulatory compounds into osteoclasts.

### Functionalized nanoparticles prevent excessive bone resorption in a medaka Rankl model

We thus assessed whether medaka macrophages also internalize functionalized CaP nanoparticles and can express sufficient levels of osteomodulatory proteins such as the osteoclast-inhibiting osteoprotegerin (Opg) from the internalized nanoparticles. During bone remodelling, Opg acts as a Rankl decoy receptor and prevents excessive bone resorption (2). Consequently, a deficiency in Opg increases bone turnover and leads to osteoporosis-like phenotypes (43, 44). On the other hand, administration of recombinant Opg reduced osteoporotic bone loss in ovariectomized or Opg-deficient mice (45, 46), confirming the bone protective effect of Opg. Opg was also shown to inhibit osteoblast apoptosis through binding to TNF-related apoptosis-inducing ligand (TRAIL) (47). Thus, Opg reduces osteoclastogenesis and promotes osteoblast survival, which makes it a potent therapeutic target for osteoporosis. Slow or intermittent release of exogenous Opg might diminish bone resorption without strongly affecting bone remodelling. In medaka, there are two *opg* genes, *opga* and *opgb*, with highly conserved cysteine-rich N-terminal domains that are important for Rankl binding, and less conserved C-terminal domains, which are shorter compared to mammalian Opg (48). In the present study, we loaded CaP nanoparticles with a plasmid expressing *opgb* under control of the *mpeg1* macrophage promoter (CaP/PEI-Cy5/*mpeg1*:*opgb*-p2a-EGFP/SiO_2_). These nanoparticles were injected into medaka larvae with normal bone development (in the absence of ectopic Rankl induction) or with induced osteoporotic lesions (after ectopic Rankl induction). The used *mpeg1* promoter restricted ectopic *opgb* expression to macrophages. Under Rankl-induced conditions, we hypothesized that a subset of macrophages internalized the functionalized nanoparticles, differentiated into osteoclasts and secreted *opgb* into the surrounding tissues. Consistently, we found that the expression of *opgb*-p2a-EGFP partially colocalized with Cy5, indicating that the majority of the injected nanoparticles efficiently delivered cargo DNA to macrophages, which subsequently expressed the functionalized plasmid. We also found that *opgb* delivered by nanoparticles did not affect bone development in the absence of Rankl induction. This is consistent with the fact that bone homeostasis at the analysed larval stages (11-13 days) does not rely on endogenous osteoclasts, which only form at later stages (21 days; 19). In stark contrast, *opgb* functionalized nanoparticles protected mineralized bone under osteoporotic conditions when excessive osteoclast formation was induced by transgenic Rankl expression. Interestingly after Rankl induction, the density of *ctsk*:nlGFP cells at the vertebral column was similar in untreated and nanoparticle-treated fish. Despite this, the mineralized matrix of neural arches was preserved after injection of Opgb nanoparticles, while it was almost completely resorbed in untreated controls. This suggests that nanoparticle-delivered Opgb did not interfere with the formation of Rankl-induced *ctsk*:nlGFP osteoclasts, but rather blocked their activity. It is likely that the levels of nanoparticle-derived Opgb were insufficient to entirely compete with transgenic Rankl and block osteoclast formation but these levels appeared sufficient to block osteoclast activity. This is consistent with a previous report showing that Opg inhibits the bone-resorbing activity of osteoclasts in giant cell tumours of bone (49).

One of the limitations of the current study is that we were not able to directly visualize the differentiation of *mpeg1*:mCherry macrophages carrying Cy5-labelled nanoparticles functionalized with *mpeg1*:*opgb*-p2a-EGFP into *ctsk*:nlGFP or *ctsk*:mCherry positive osteoclasts. This was not possible as emission wavelengths of the four fluorescence reporters overlapped. Nevertheless, our study provides strong evidence that functionalized CaP nanoparticles are stable in medaka larvae and are efficiently taken up by macrophages that differentiate into osteoclasts under osteoporotic conditions. After targeted injection into tissue adjacent to bone, these nanoparticles served as efficient vehicles to deliver Opgb at sufficient levels to block osteoclast activity and protected bone from Rankl-induced osteoporotic insult.

## Data availability statement

The original contributions presented in the study are included in the article/**Supplementary Material**. Further inquiries can be directed to the corresponding author.

## Ethics statement

The animal study was reviewed and approved by National University of Singapore IACUC.

## Author contributions

NI, VS, ME and CW contributed to conception and design of the study. NI, VS and KK generated the data. NI, VS, KK, ME and CW analyzed the data. NI performed the statistical analysis. NI, VS and KK wrote the first draft of the manuscript. VS, ME and CW revised and edited the manuscript. All authors contributed to manuscript revision, read, and approved the submitted version. All authors contributed to the article and approved the submitted version.
