# Supplementary material for: Functionalized calcium phosphate nanoparticles to direct osteoprotegerin to bone lesion sites in a medaka (Oryzias latipes) osteoporosis model
Source: Front Endocrinol (Lausanne). 2023 Feb 22;14:1101758. doi: 10.3389/fendo.2023.1101758 (PMC9992893; doi:10.3389/fendo.2023.1101758)
Supplement: Supplementary file 1 [file DataSheet_1.docx]

**Supplementary Material**

**Supplementary Figure S1. Map of *mpeg1:opgb*-p2a-EGFP plasmid**. The pI-*Sce*I vector backbone contains a *mpeg1* promoter, Tol2 and *I-SceI* sites for enhanced genomic integration, and an Ampicillin resistance gene (AmpR). The insert containing the *opgb* coding sequence (cds) followed by a self-cleaving p2a peptid sequence and EGFP-SV40A was introduced after *EcoRI/ApaI* double digestion.

**Supplementary Figure S2. Mosaic EGFP expression in medaka larva injected with *mpeg1:opgb*-p2a-EGFP plasmid at one cell stage. (A)** Lateral view of medaka larva at 14 dpf with mosaic EGFP expression in the middle of the trunk. **(A’)** High magnification view of area indicated as box in (A). Arrowheads indicate individual EGFP positive cells. Note rows of autofluorescent pigment cells located in the dorsal- and ventral-most regions of the trunk.

**Supplementary Figure S3. *Col10a1:*nlGFP positive osteoblast progenitors do not internalize functionalized CaP nanoparticles. (A-A”’)** Lateral views of caudal fin ray of *col10a1*:nlGFP transgenic medaka at 9 dpf, 5 hours after injection of CAP/PEI-Cy5/pDNA-mCherry/SiO_2_ functionalized nanoparticles into the fin. Osteoblast progenitors express *col10a1*:nlGFP (A), and cells that internalized functionalized CaP nanoparticles express mCherry (A’). There is no colocalization of nlGFP and Cy5 (A”), or nlGFP and mCherry (A’”). **(B-B”)** Virtual cross sections obtained from confocal stacks from samples shown in A. Arrows mark Cy5-labelled nanoparticles that lead to mCherry expression but are not colocalized with *col10a1*:nlGFP cells. 16 out of 16 analysed embryos showed this phenotype. Scale bars = 10 µm (A), 4 µm (B).

**Supplementary Figure S4. *osx:GFP* positive premature osteoblasts do not internalize functionalized CaP nanoparticles. (A-A”’)** Lateral views of caudal fin ray of *osx:GFP* transgenic medaka larva at 14 dpf, 5 hours after injection of CAP/PEI-Cy5/pDNA-mCherry/SiO_2_ functionalized nanoparticles into the fin. Premature osteoblasts express *osx:GFP* (A), and cells that internalized functionalized CaP nanoparticles express mCherry (A’). There is no colocalization of GFP and Cy5 (A”), or GFP and mCherry (A’”). **(B-B”)** Virtual cross sections obtained from confocal stacks as shown in A. Arrows mark Cy5-labelled nanoparticles that lead to mCherry expression but are not colocalized with *osx:GFP* cells. 12 out of 12 analysed embryos showed this phenotype. Scale bars = 15 µm (A) and 5 µm (B).

**Supplementary Figure S5. Injection of water triggers macrophage recruitment to injection site in caudal fin. (A, B)** *mpeg1:mCherry* positive macrophages (blue) at 1 hpi (A) and 18 hpi (B). **(A’, B’)** Merged images showing brightfield and mCherry at 1 hpi (A’) and 18 hpi (B’).

**Supplementary Figure S6. Additional examples for protection of mineralized vertebral arches from Rankl-induced resorption by nanoparticle-derived *mpeg1:opgb*:GFP. (A-D)** For control, *ctsk*:mCherry/*rankl*:*HSE*:CFP transgenic larvae (without injection of Cy5-labeled CaP nanoparticles) were heat-shocked at 9 dpf for Rankl induction and bone mineralization was evaluated using calcein staining at 12 dpf. Rankl induction resulted in formation of *ctsk*:mCherry expressing osteoclasts that resorbed neural arches (calcein stain; empty arrowheads). **(E-H)** Cy5-labelled CaP nanoparticles functionalized with *mpeg1:opgb-*p2a-EGFP plasmid were injected into trunk muscle of *ctsk*:mCherry/*rankl*:*HSE*:CFP transgenic larvae 1 hour after Rankl induction at 9 dpf and bone mineralization was analyzed using calcein staining at 12 dpf. Neural arches in Rankl-induced larvae with *ctsk*:mCherry expressing osteoclasts remained largely intact after treatment with Opgb delivered by CaP nanoparticles (calcein stain; arrowheads).

**Supplementary Movies**

**Movie 1.** Macrophages (blue) are recruited to the injection site and take up nanoparticles (red) in the caudal fin. *ctsk*:nlGFP-expressing cells in green.

<https://www.dropbox.com/s/pwiwfgvzk0f9fi7/movie4.mp4?dl=0>

**Movie 2.** Rankl-induced macrophages (blue) take up nanoparticles (red) and then differentiate into osteoclasts (green) in muscle.

<https://www.dropbox.com/s/6ado2ertbvwflha/movie5.mp4?dl=0>
