# Supplementary material for: Functionalized calcium phosphate nanoparticles to direct osteoprotegerin to bone lesion sites in a medaka (Oryzias latipes) osteoporosis model
Source: Front Endocrinol (Lausanne). 2023 Feb 22;14:1101758. doi: 10.3389/fendo.2023.1101758 (PMC9992893; doi:10.3389/fendo.2023.1101758)
Supplement: Supplementary file 2 [file Table_1.docx]

**Supplementary Table 1. List of transgenic medaka lines used in this study.**

| Line | Description | Source |
| --- | --- | --- |
| *rankl*:*hse*:CFP | Allows ubiquitous induction of Rankl upon heat-shock | To et al. (2012) |
| *mpeg1*:mCherry | Marks macrophages, osteoclast progenitors | Phan et al. (2020) |
| *ctsk*:nlGFP, *ctsk*:mCherry | Marks osteoclasts | To et al. (2012) |
| *col10a1*:nlGFP | Marks osteoblast progenitors, premature osteoblasts, and chondrocytes | Renn et al. (2013) |
| *osx*:GFP | Marks premature osteoblasts | Renn & Winkler (2012) |

**Supplementary Table 2. Primers used to generate *mpeg1:opgb*-p2a-EGFP.**

| **Name of oligo** | **Sequence 5’ to 3’** | **Application** |
| --- | --- | --- |
| EcoRI_Kozak_opg_F | GGGGAATTCCCACCATGACAGTGCTTTACC | Plasmid cloning |
| opg_P2A_R | CAGCAGGCTGAAGTTTGTAGCTGGAAAAATCAAGCTAC | Plasmid cloning |
| P2A_opg_F | GTAGCTTGATTTTTCCAGCTACAAACTTCAGCCTGCTG | Plasmid cloning |
| ApaI_GFP_R | AAAGGGCCCGCGGCCGCGAATTAAAAAA | Plasmid cloning |
| Seq_mpeg_F | TCCCTTTGTTTCCCCATGACA | Plasmid sequencing |
| Seq_opg_R | GCCTGGTTCACACTTCTTGC | Plasmid sequencing |
