# Supplementary figures and images for: Functionalized calcium phosphate nanoparticles to direct osteoprotegerin to bone lesion sites in a medaka (Oryzias latipes) osteoporosis model
Source: Front Endocrinol (Lausanne). 2023 Feb 22;14:1101758. doi: 10.3389/fendo.2023.1101758 (PMC9992893; doi:10.3389/fendo.2023.1101758)

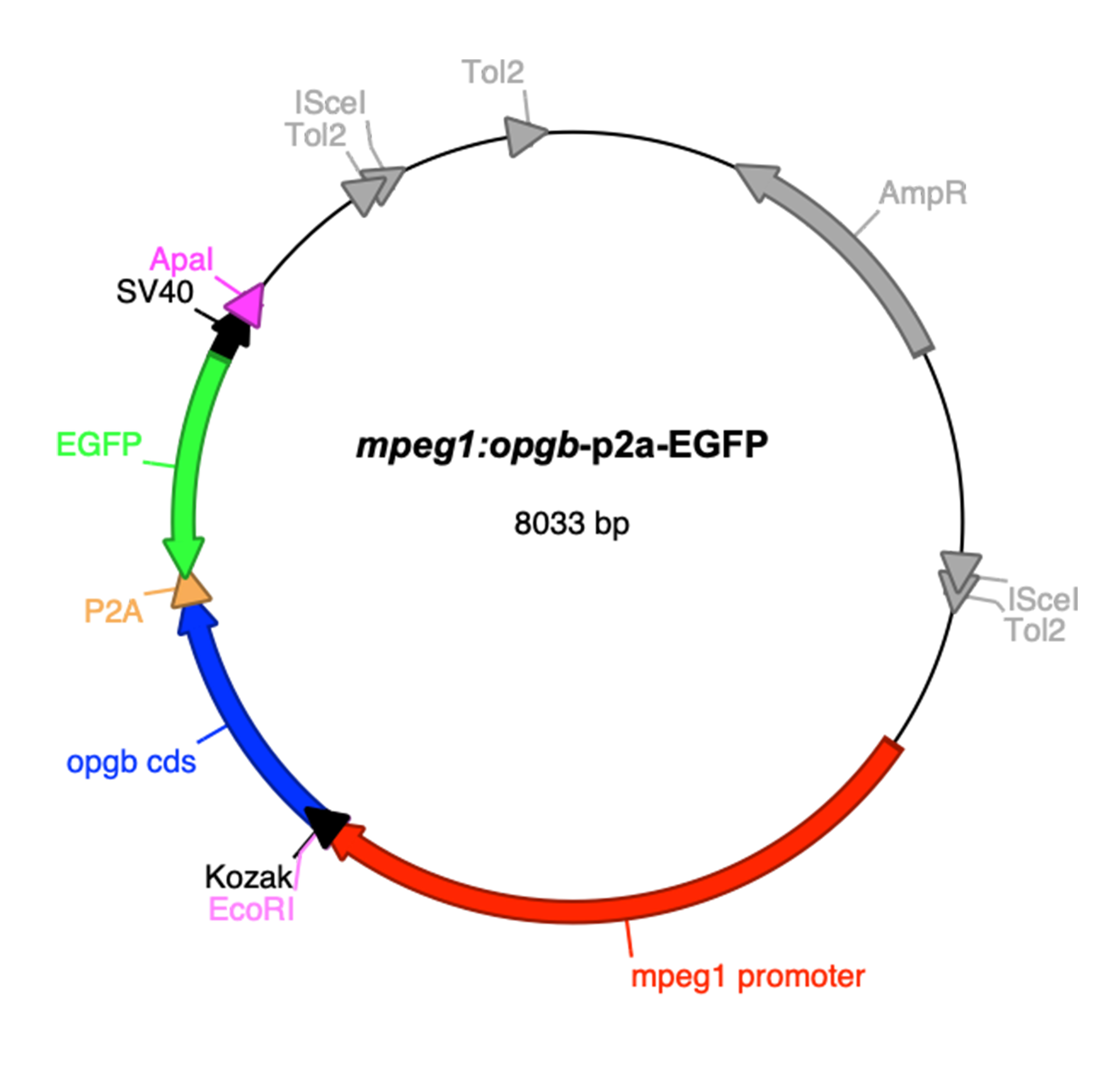

Supplement: Supplementary file 3 [file Image_1.jpeg]

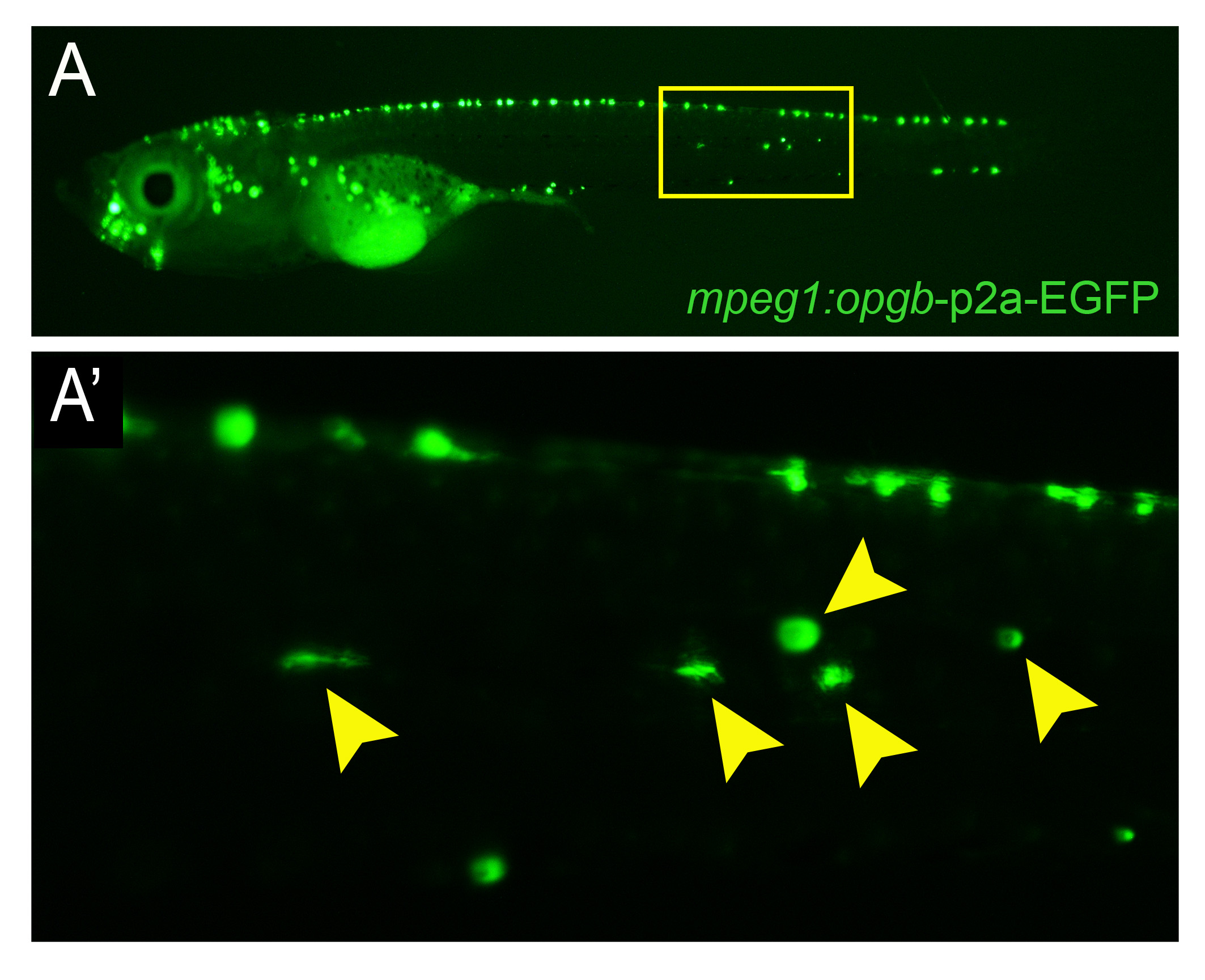

Supplement: Supplementary file 4 [file Image_2.jpeg]

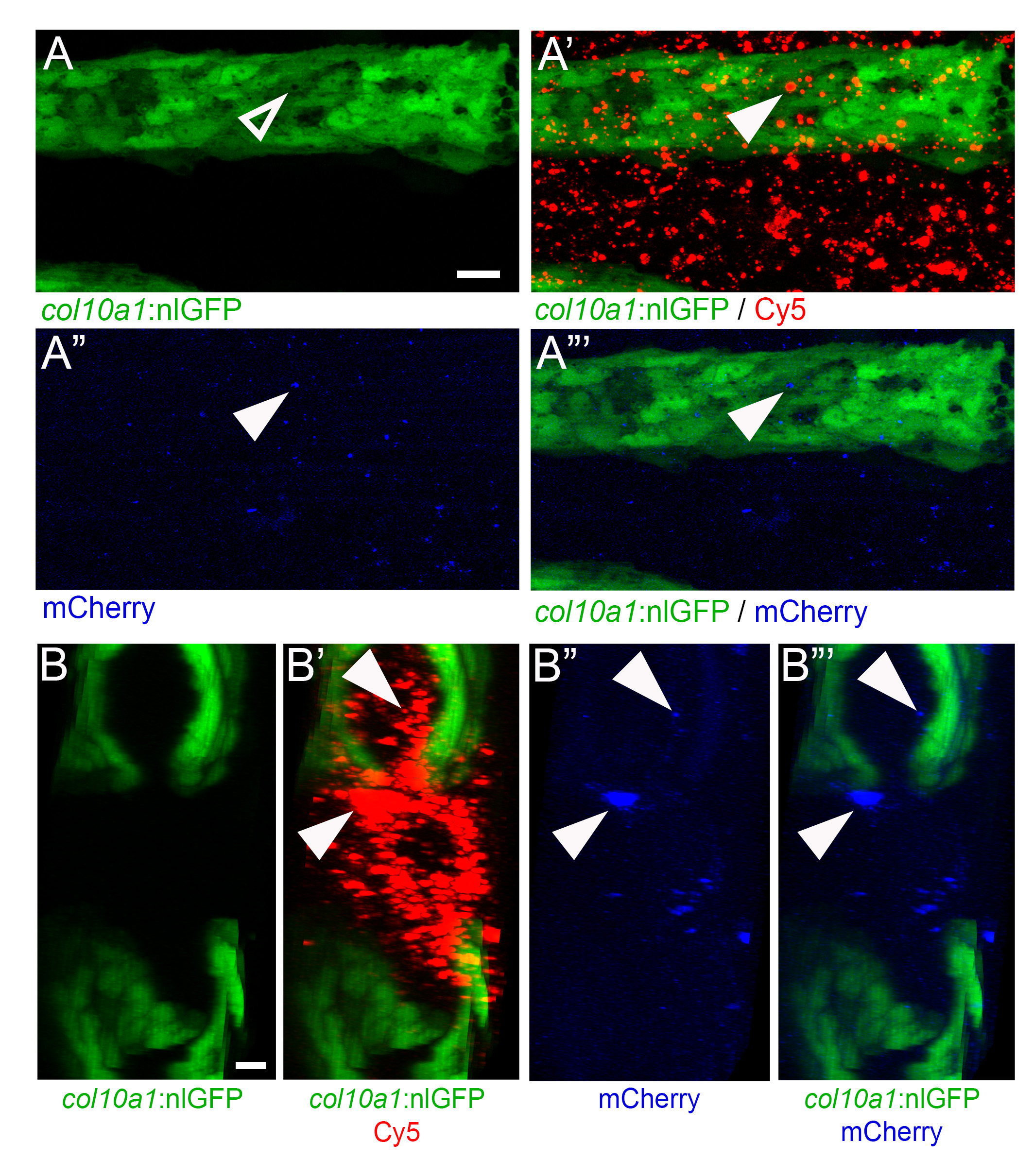

Supplement: Supplementary file 5 [file Image_3.jpeg]

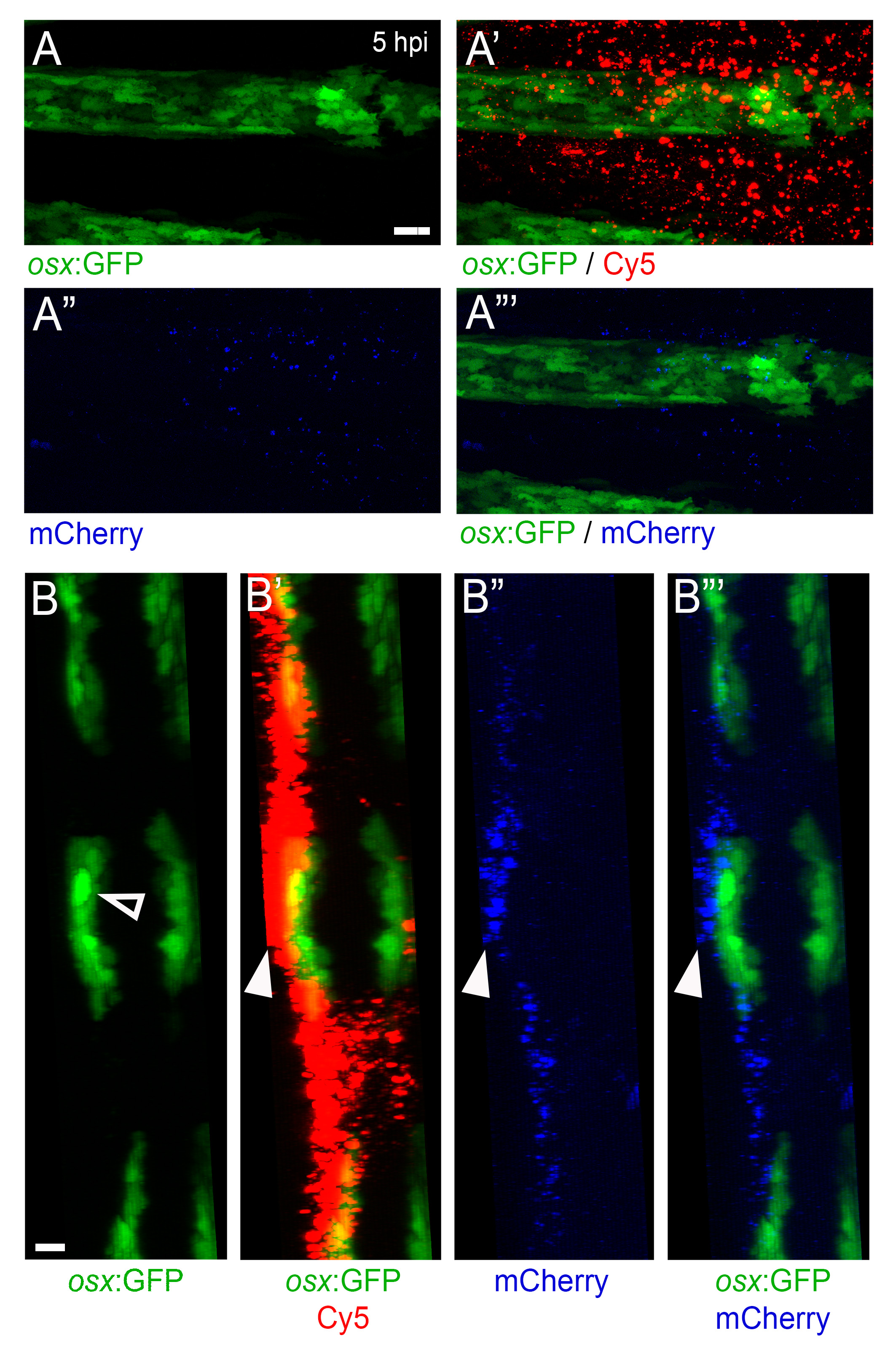

Supplement: Supplementary file 6 [file Image_4.jpeg]

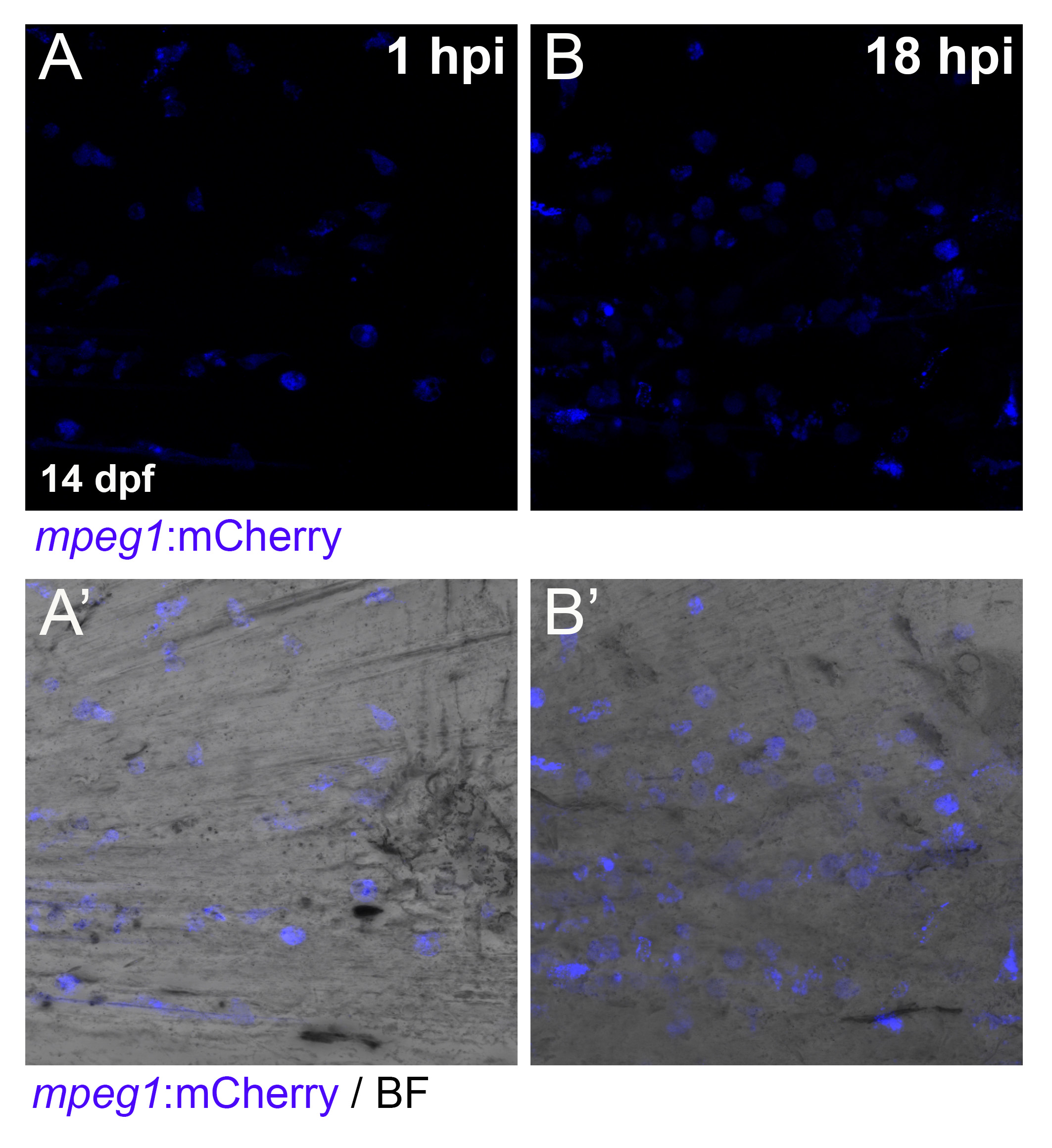

Supplement: Supplementary file 7 [file Image_5.jpeg]

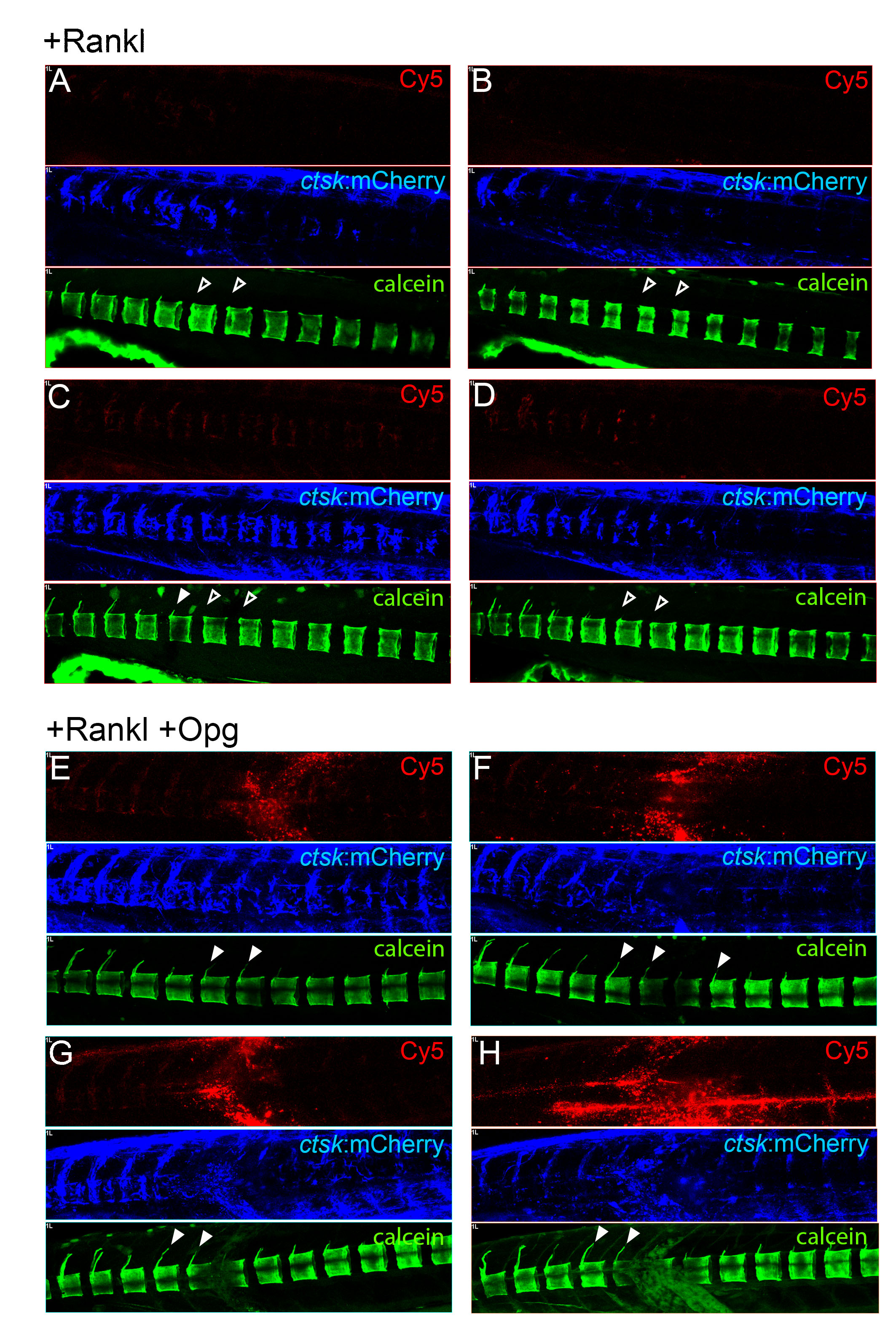

Supplement: Supplementary file 8 [file Image_6.jpeg]
